# Adaptive evolution of a generalist parasitoid: implications for the effectiveness of biological control agents

**DOI:** 10.1111/eva.12081

**Published:** 2013-08-05

**Authors:** Francisca A Zepeda-Paulo, Sebastián A Ortiz-Martínez, Christian C Figueroa, Blas Lavandero

**Affiliations:** 1 Facultad de Ciencias, Instituto de Ciencias Ambientales y Evolutivas, Universidad Austral de ChileValdivia, Chile; 2 Laboratorio de Interacciones Insecto-Planta, Instituto de Biología Vegetal y Biotecnología, Universidad de TalcaTalca, Chile

**Keywords:** *Aphidius ervi*, biological control, divergent selection, infectivity, local adaptation, parasitoid, phenotypic plasticity, virulence

## Abstract

The use of alternative hosts imposes divergent selection pressures on parasitoid populations. In response to selective pressures, these populations may follow different evolutionary trajectories. Divergent natural selection could promote local host adaptation in populations, translating into direct benefits for biological control, thereby increasing their effectiveness on the target host. Alternatively, adaptive phenotypic plasticity could be favored over local adaptation in temporal and spatially heterogeneous environments. We investigated the existence of local host adaptation in *Aphidius ervi*, an important biological control agent, by examining different traits related to infectivity (preference) and virulence (a proxy of parasitoid fitness) on different aphid-host species. The results showed significant differences in parasitoid infectivity on their natal host compared with the non-natal hosts. However, parasitoids showed a similar high fitness on both natal and non-natal hosts, thus supporting a lack of host adaptation in these introduced parasitoid populations. Our results highlight the role of phenotypic plasticity in fitness-related traits of parasitoids, enabling them to maximize fitness on alternative hosts. This could be used to increase the effectiveness of biological control. In addition, *A. ervi* females showed significant differences in infectivity and virulence across the tested host range, thus suggesting a possible host phylogeny effect for those traits.

## Introduction

Agricultural intensification under the current food production scenario is causing novel ecological and evolutionary changes in agroecosystems globally, mainly through the escalation of biotic interactions and the introduction of new pest species (Zimmerer 2010; Thrall et al. 2011). Traditionally, pest management has been largely based on the intensified use of chemical control. However, the use of insecticides has triggered several undesired effects including the continued evolution of insecticide resistance, the bioaccumulation of pesticides at different trophical levels, and the inherent risk to human health, among others (van Lenteren 2000; Bale et al. 2008). Hence, current efforts in pest management are aimed to reduce the environmental risks and to increase the efficiency of resource-use in agriculture (Thrall et al. 2011). In this context, eco-evolutionary research on biological control agents is important to understand how to increase the efficacy and safety of biological pest control (Hufbauer and Roderick 2005; Bale et al. 2008; Henry et al. 2010; Vorsino et al. 2012).

During the past 120 years, more than 5000 biological introductions of approximately 2000 different species of natural enemies of arthropods have been carried out to control the population density of pest species (i.e., classical biological control) (Roderick and Navajas 2003; van Lenteren et al. 2006a; Cock et al. 2010). However, only 16% of all those introductions have established and resulted in the successful control of the target pest (Caltagirone and Doutt 1989; Bellows 2001; Roderick and Navajas 2003; Grandgirard et al. 2006). This low success rate of man-made introductions of biological controllers has been explained, in part, as the result of (i) the introduction of agents poorly adapted to local environmental conditions, (ii) the evolution of nontarget interactions with new hosts and other guild interactions such as competition and intraguild predation (Messing et al. 2006; Gariepy and Messing 2012), and (iii) the persistence of undesirable or maladaptive traits in the introduced populations of natural enemies (Hufbauer 2002; Thrall et al. 2011). The latter is a direct consequence of the low number of propagules (i.e., founder effect) and the genetic bottlenecks during the introduction process of a control agent. Indeed, during the introduction of natural enemies, the adaptive genetic variation could be randomly reduced, thus limiting the adaptive responses of the new populations and thereby decreasing the performance on their target hosts (Hufbauer et al. 2004a; Hufbauer and Roderick 2005; Vorsino et al. 2012). Nevertheless, the implications of the adaptive potential of the natural enemy populations selected for introduction and the recent coevolutionary history between the natural enemies and their host species have been typically ignored by pest control strategies in general (including classical biological control) (Henry et al. 2010). In fact, there is a lack of empirical evidence on the significance of the evolutionary and adaptive processes underpinning the success of biological control, which appears particularly evident with control agents that are mass-reared before being released into agricultural systems (Roderick and Navajas 2003; Henry et al. 2010; Vorsino et al. 2012).

Parasitoid Hymenoptera are highly specialized natural enemies commonly used as biocontrol agents in agro-ecosystems (Godfray 1994). Although the mechanisms involved in the adaptive evolution of parasitoid populations are still poorly understood, some authors have suggested that the main factor contributing to the divergence and specialization of parasitoid populations could be the close relationship they establish with their insect hosts, representing a potential for divergent selection of parasitoid populations using different hosts (Stireman et al. 2006; Abrahamson and Blair 2008; Feder and Forbes 2010). The evolution of highly adaptive races to their host plants (e.g., host race formation and ecological speciation in insects) has been well studied in herbivorous insects (Drès and Mallet 2002; Funk 2010). This kind of adaptive evolution is also expected to occur in insect parasitoids. Whenever parasitoid populations use different environments, divergent natural selection could promote local adaptation in different populations, conferring greater fitness on their natal environment compared to other non-natal environments (Kawecki and Ebert 2004). Furthermore, in parasitoids, different hosts can act as divergent selection agents, which could differentially affect behavioral traits associated with host selection (i.e., infectivity in parasitoids), as well as physiological incompatibilities associated with the susceptibility of the hosts (i.e., virulence in parasitoids), thus resulting in adaptations to a certain host (Mackauer et al. 1996; Antolin et al. 2006). Different experimental evolutionary studies have demonstrated the existence of the highly adaptive potential of parasitoid populations (Henry et al. 2008; Dion et al. 2011). Host-associated genetic differentiation in natural populations of parasitoids has supported the hypothesis of ‘sequential radiation’ raised by Abrahamson and Blair (2008). This hypothesis proposes that the diversification of herbivorous insects through the adaptation to new host plants and the formation of ecological races (i.e., host races) represents new resources available for the parasitoids associated with those herbivorous races, which in turn promote the adaptation and diversification of parasitoid populations (Stireman et al. 2006; Forbes et al. 2009). Therefore, understanding the evolutionary mechanisms underpinning host adaptation in parasitoid populations could improve current strategies for biological control. For instance, finding preadapted biological control agents for the target hosts in their native range could imply an increased chance that this parasitoid population would become established on a certain target pest. This would foster greater efficiency in host location and the ability to overcome host resistance, resulting in higher rates of population increase (Hufbauer and Roderick 2005). However, host adaptation could also involve a cost associated with reducing the phenotypic plasticity of parasitoid populations, when the preferred host is rare or not available and then populations may be maladapted to the current host (Hufbauer 2002; Antolin et al. 2006). Alternatively, phenotypic plasticity may dampen the effects of selection regimes (different hosts) by allowing individuals to adapt to alternate hosts (Crispo 2008). Adaptive plasticity reduces the effective magnitude of environmental shifts, facilitating population persistence (Chevin and Lande 2009) and thereby increasing ecological resilience at a regional scale (Laliberté and Tylianakis 2010).

The parasitoid *Aphidius ervi* (Haliday) (Hymenoptera: Braconidae; Aphidiinae) is commonly used in biological control in agriculture and possibly one of the best-studied parasitoid model systems in ecology and evolution (Henry et al. 2010). This endoparasitoid is a solitary koinobiont that parasitizes several Macrosiphinae species in their source region (Eurasia) (Starý et al. 1993). In Europe, *A. ervi* is frequently recorded on legume aphids, such as the pea aphid *Acyrthosiphon pisum* (Harris) (Tomanovic et al. 2009). When introduced in North America, the host diversity of *A. ervi* was presumably more restricted (Bilodeau et al. 2013). In Chile (South America), *A. ervi* was introduced about 35 years ago from France, as part of a classical biological control program of the grain aphid *Sitobion avenae* (Fabricius), one of the most important cereal pests in Chile (Zuñiga et al. 1986a). At present, *A. ervi* has proven to be highly efficient in controlling aphids on legume and cereal crops and even becoming the most common parasitoid of *S. avenae* (up to 75% in the field) (Gerding and Figueroa 1989; Gerding et al. 1989; Zuñiga 1990; Starý et al. 1993, 1994). Interestingly, this latter situation is not commonly observed in Europe, where other species have a greater relevance than *A. ervi* in controlling cereal aphids (Cameron et al. 1984; Kos et al. 2009).

Consequently, the aim of this study was to investigate the adaptation of introduced populations of the parasitoid *A. ervi* to different aphid hosts in Chile. In order to determine local host adaptation, we tested the hypothesis that each parasitoid population had a higher fitness on its own host (natal host) than on other non-natal aphid hosts. For this, we conducted reciprocal transplant experiments and studied different traits associated with the infectivity and virulence (fitness-related traits) of different host-associated parasitoid populations across different aphid hosts.

## Materials and methods

### Parasitoids and aphids

Aphids were collected from fields of legumes and cereals in two different geographic zones in Chile: Region del Maule (S 35°24′, W 71°40′) and Region de Los Rios (S 39°51′, W 73°7′). Parasitoid individuals were obtained by collecting and rearing aphids from the field. This method allowed an accurate determination of the aphid species from which parasitoid individuals were obtained. The pea aphid *Acyrthosiphon pisum* complex was separately studied including the two different host races present in Chile (Peccoud et al. 2008). The alfalfa race of *A. pisum* (APA) was sampled on alfalfa fields (*Medicago sativa* L.) and the pea race (APP) on pea orchards (*Pisum sativum* L.). Furthermore, the cereal aphids studied included the grain aphid *Sitobion avenae* (SA) and the bird cherry-oat aphid *Rhopalosiphum padi* (L.) (RP), both sampled on wheat (*Triticum aestivum* L.) and oat (*Avena sativa* L.) fields.

All aphids were reared under controlled laboratory conditions on the same host plant species from which they were collected in the field, which allowed the continued reproduction of aphids and parasitoids (20°C, 50–60 RH, D16/N8 of photoperiod). Aphids were kept until the appearance of mummies (i.e., aphid exoskeletons containing the parasitoid pupae) and later emergence of parasitoids occurred. Each parasitoid individual that emerged from a collected mummy was determined using a taxonomic key described by Starý (1995). All *A. ervi* individuals were reared on the same host aphid species from which they emerged (natal host) in the laboratory. Thus, four different *A. ervi* populations were established: (i) *A. ervi* population from *A. pisum*-alfalfa race; (ii) *A. ervi* population from *A. pisum*-pea race; (iii) *A. ervi* population from *S. avenae*; and (iv) *A. ervi* population from *R. padi*. In order to limit the loss of genetic diversity as a consequence of genetic drift, all rearing populations (i.e., from which experimental parasitoid individuals were obtained) comprised a high number of parasitoid individuals collected in the field (>300 individuals). Only the first six parasitoid generations were used for further performance experiments. Female experimental parasitoids were obtained as mummies from rearing colonies, then transferred to a plastic box (10 × 20 × 15 cm) before emergence, supplied with water, and diluted honey (10%) for feeding and male parasitoids for mating. Female parasitoids up to 2 days of age were used in the experiments. Since endosymbiotic bacteria such as *Hamiltonella defensa* have been shown to confer resistance to *A. ervi* parasitism in the *A. pisum* aphid (Oliver et al. 2010), only aphid clones free of most common secondary bacteria described for aphids (*Spiroplasma spp*., *Serratia symbiotica*, *Regiella insecticola*, *Rickettsia spp*. y *Hamiltonella defensa*) were used for maintenance and experimentation. The detection of secondary bacteria in aphids was carried out by PCR of a 16S rDNA mitochondrial region (see Tsuchida et al. 2002 for further details), using the primary endosymbiont of aphids, *Buchnera aphidicola,* as a positive control (Oliver et al. 2010).

### Reciprocal transplant experiments

To study the response of parasitoids to different selection agents (different host aphid species), a reciprocal transplant experiment was conducted to determine the infectivity and virulence of parasitoid females to their natal hosts (i.e., the aphid species on which parasitoids were collected in the field) and non-natal hosts. This type of experiment has proven to be useful in the detection of adaptive patterns, studying the mean fitness shown by a set of populations or demes through a set of experimental habitats, and allowing the direct testing of the role of a particular environmental factor as a divergent selection agent (Kawecki and Ebert 2004).

### Parasitoid infectivity

Parasitoid infectivity was described through the recording of a suit of behaviors. Previous observations and published studies (Wang and Keller 2002; Araj et al. 2011) were revised to choose relevant behavioral traits of the parasitoid females. The following behaviors were considered for analyses:

1. Sting, the insertion of the female ovipositor into the host's body, characterized by the curving of the abdomen in a forward position between the third pair of legs;
2. Attack, similar to *sting* but with no successful insertion of the ovipositor into host's body;
3. Walking, a persistent walking movement all over the experimental arena.

Infectivity experiments were carried out on four aphid hosts (APA, APP, SA, and RP) testing all four parasitoid populations. Experimental arena consisted of a modified glass Petri dish (2 cm. diameter) containing one aphid nymph of the second to third instar of each aphid host onto a small piece of leaf (from the host plant where each aphid species was reared). Second to third instars were chosen as they perform less defensive behaviors (e.g., kicking) and represent a high-quality resource for *A. ervi*, being normally preferred over other nymphal stages (Henry et al. 2008). After 5 min of settling of the aphid on the leaf, one single-mated naive female parasitoid per assay was placed inside the experimental arena and behaviors were recorded during 10 min. Female parasitoids were used only once. Behavioral observations were done under an Optika ST-155 (10×) compound microscope with a diffuse cold light source under the experimental arena. Each test combination (parasitoid population X aphid host = 16 combinations) was repeated at least 10 times, renewing the experimental arena for every new test (Petri dish, plant, aphid, and test parasitoid). The proportion of time spent for each of the three behavioral traits was estimated, as well as the frequency of sting and attack and the time to first ‘sting’ and ‘attack’ using software etholog v.2.2.5 (Ottoni 2000). To increase accuracy, all tests were recorded with a high-resolution video camera (C-mount SZNCTV1/2).

### Parasitoid virulence

Virulence experiments were carried out on the four aphid hosts, but only three parasitoid populations (APA, APP, and SA) were tested, because *R. padi* proved to be a low-quality host for *A. ervi* (see results and discussion). A total of 300 female parasitoids were assayed (25 replicate for each treatment). Each assay was conducted in an experimental arena (5-cm-diameter glass Petri dish), containing ten nymphs from second to third instars. Subsequently, a single previously mated, naive female parasitoid was placed into the experimental arena (each female was used only once) and observed until the female parasitoid made a successful oviposition in each aphid (i.e., female parasitoid inserting ovipositor for a period longer than 5s; Desneux et al. 2009). After each successful oviposition, stung aphid nymphs were removed from the experimental arena, transferred to their host plant, and confined to a clip cage (Noble 1958). The experiment ended after 10 min or after ten aphid nymphs were stung. For the following ten days, the number of mummies formed was recorded and the number and sex of the emerging parasitoids was registered. For virulence, parasitism rate, survival of progeny, and productivity (i.e., product of the fecundity and survival of parasitoids) were recorded. Developmental time and the sex ratio of the progeny were also estimated as proxies of parasitoid fitness. In hymenopteran parasitoids, the sex ratio is under direct behavioral control of the parasitoid females, being an indicator of adaptive host selection, where fertilized eggs could be preferentially allocated to higher quality hosts (Charnov et al. 1981; Godfray 1994). The parasitism rate was estimated as the mean number of parasitized aphids (number of mummies produced from stung nymphs), divided by the total number of aphid survivors plus the number of mummies formed (Antolin et al. 2006). The survival of the progeny was estimated as the mean proportion of parasitoids which emerged from mummies formed (number of parasitoids emerged/number of mummies formed) (Henry et al. 2010). Productivity was estimated as the mean number of parasitoids that emerged from stung aphids (number of mummies formed plus aphid survivors) (Antolin et al. 2006; Dion et al. 2011). Productivity is considered a good measure of parasitoid virulence given that it includes both fecundity (parasitism rate) and survival of the parasitoid progeny, and thus, it becomes a good proxy of parasitoid fitness (Roitberg et al. 2001; Antolin et al. 2006). Developmental time was estimated as the time from oviposition to the emergence of an adult parasitoid. The sex ratio was calculated as the proportion of males present in the progeny of a single female parasitoid in each assay.

### Statistical analysis

Significant differences in infectivity between natal and non-natal hosts (four levels) were analyzed for each parasitoid population utilizing generalized linear mixed models (GLMMs) (Bolker et al. 2009). The proportion of time spent for the relevant behaviors was analyzed assuming a binomial error and a logit-link function for proportional data. The frequency of attack and sting was analyzed using a Poisson error and a log-link function for frequency data, whereas the time to the first ‘sting’ and ‘attack’ was compared for each parasitoid population tested using survival analyses (Kaplan-Meier estimates) (Hosmer and Lemeshow 1999), with the *survival* packages (Therneau 1999; Fox and Carvalho 2012) implemented in *R Commander* (Fox 2005). The effect of the assayed hosts (four levels) and the parasitoid population (three levels) (fixed factors), as well the interaction between these two independent variables on each dependent variable (virulence), was analyzed using generalized linear mixed models (GLMMs). The dependent variables: parasitism rate, survival of progeny, productivity, and sex ratio were analyzed assuming a binomial error and a logit-link function. The developmental time was analyzed using a Poisson error and a log-link function. The random factors included in the GLMMs corresponded to a temporal block (twenty-six levels) and the generation of parasitoids assayed (six levels). However, as the generation factor did not show any variation within the models as random factors, they were therefore not included in the final analysis. All GLMMs were conducted using *lme4* package (Bates et al. 2010) in R 2.15.1 (R Development Core Team 2008). In addition, the GLMMs with overdispersion were analyzed by fitting the model, including a random factor at the individual level (Elston et al. 2001; Browne et al. 2005). The different models were compared using the Akaike criterion and performing an anova in the *car* package (Fox et al. 2009). Pairwise comparisons were developed using ‘Tukey’ tests, correcting for multiple comparisons by the ‘single-step’ method using *Multcomp* package (Hothorn et al. 2008).

## Results

### Infectivity on natal and non-natal hosts

Variation of infectivity was measured by the observation of a set of behaviors carried out by female parasitoids when different aphid hosts were offered. The two most important and relevant behaviors were *attack* and *sting*.

#### Attack behavior

**Figure 1:**
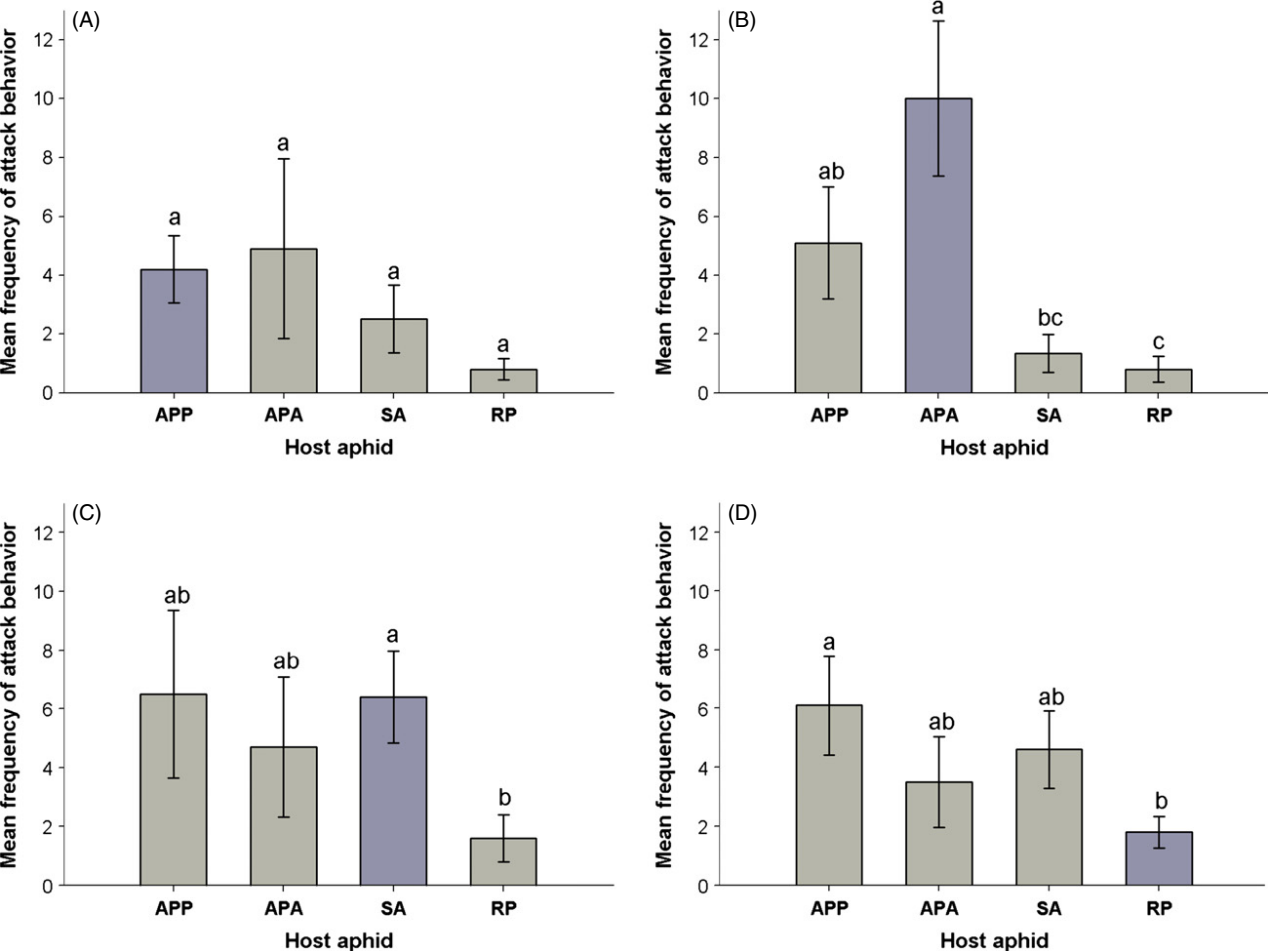
Mean frequency of *attack* of each parasitoid population on natal host (dark bars) and on non-natal hosts (light bars) (Mean ± SE). Different letters indicate significant differences expressed per parasitoid population assayed on the four hosts tested; *Acyrthosiphon pisum*-pea race (APP), *Acyrthosiphon pisum*-alfalfa race (APA), *Sitobion avenae* (SA), and *Rhopalosiphum padi* (RP). Figure: Parasitoid collected from (A) *A. pisum*-pea race; (B) *A. pisum*-alfalfa race; (C) *S. avenae* and (D) *R. padi*.

**Figure 2:**
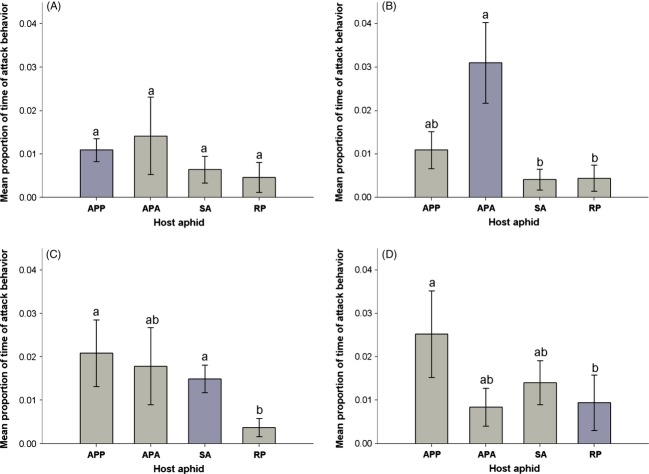
Mean proportion of time spent attacking natal (dark bars) and non-natal hosts (light bars) of each parasitoid population (Mean ± SE). Different letters indicate significant differences expressed per parasitoid population assayed on the four hosts tested; *Acyrthosiphon pisum*-pea race (APP), *Acyrthosiphon pisum*-alfalfa race (APA), *Sitobion avenae* (SA), and *Rhopalosiphum padi* (RP). Figure: Parasitoid collected from (A) *A. pisum*-pea race; (B) *A. pisum*-alfalfa race; (C) *S. avenae* and (D) *R. padi*.

The variables frequency and proportion of time spent attacking showed a significant effect for the assayed host in the parasitoid populations from the *A. pisum*-alfalfa race, *S. avenae* and *R. padi*, with no significant differences for the parasitoids from the *A. pisum*-pea race (Table 1). The *A. pisum*-alfalfa parasitoid population showed a high frequency and proportion of time spent attacking on their natal host, when compared to other non-natal hosts. However, this was only statistically significant compared to when *S. avenae* and *R. padi* were offered as hosts (Figs 1 and 2). In contrast, *R. padi*-originated populations had lower values for the natal when compared to other non-natal hosts tested. Significant differences were also found for the time to the first *attack* for the parasitoid population from the *A. pisum*-pea race (Likelihood ratio test = 9.11, *P* = 0.028; logrank test = 9.97, *P* = 0.018; df = 3) and for the *S. avenae* population (Likelihood ratio test = 8.57, *P* = 0.035; logrank test = 9.11, *P* = 0.028; df = 3) (Table 2). Contrastingly, no significant differences for the time to the first *attack* of hosts was found for the *A. pisum*-alfalfa race parasitoid population (Likelihood ratio test = 4.7, *P* = 0.195; logrank test = 5.04, *P* = 0.169; df = 3) nor for the *R. padi*-originated parasitoid population (Likelihood ratio test = 5.97, *P* = 0.113; logrank test = 6.33, *P* = 0.096; df = 3) (Table 2).

**Table 1:** Generalized linear mixed models performed for each behavioral variable tested on hosts coming from different parasitoid populations

Models	Parasitoid population											

	*Acyrthosiphon pisum*-alfalfa race			*Acyrthosiphon pisum*-pea race			*Sitobion avenae*			*Rhopalosiphum padi*		
			
	df	χ^2^	*P*-value	df	χ^2^	*P*-value	df	χ^2^	*P*-value	df	χ^2^	*P*-value
Proportion of time spent												
Attack	3	19.55	<0.0001	3	5.88	0.1172	3	12.72	0.0052	3	9.45	0.0238
Sting	3	26.11	<0.0001	3	3.71	0.2944	3	31.64	<0.0001	3	7.54	0.0562
Walking	3	8.15	0.0428	3	1.59	0.6606	3	3.731	0.292	3	4.60	0.2033
Frequency												
Attack	3	25.89	<0.0001	3	6.57	0.0868	3	10.41	0.0154	3	10.64	0.0137
Sting	3	22.01	<0.0001	3	1.61	0.656	3	33.10	<0.0001	3	5.58	0.1334

**Table 2:** Kaplan–Meier estimator of the mean time to first *attack* and *sting* and standard errors (Mean ± SE) computed for each parasitoid population assayed on their natal and non-natal hosts

Parasitoid population	Assayed host	Time to first attack	Time to first sting
*A. pisum*-alfalfa race	APA	**78.33 ± 0.1581**	**38,54 ± 0,269**
	APP	262.01 ± 0.1581	54.81 ± 0.1581
	SA	324.86 ± 0.166	85.64 ± 0.166
	RP	280.7 ± 0.1549	238.04 ± 0.3581
*A. pisum*-pea race	APP	**132.5 ± 0.1581**	**156.8 ± 0.1581**
	APA	583 ± 0.1549	355.08 ± 0.1449
	SA	150.59 ± 0.1581	339.9 ± 0.1581
	RP	394.9 ± 0.1581	*
*R. padi*	RP	**234.7 ± 0.1581**	**212.34 ± 0.1581**
	APA	284.4 ± 0.1581	79.1 ± 0.1549
	APP	50.78 ± 0.1581	163.68 ± 0.1581
	SA	94.72 ± 0.1581	128 ± 0.1581
*S. avenae*	SA	**77.4 ± 0.1581**	**114.06 ± 0.1581**
	APA	80.76 ± 0.1581	187.65 ± 0.1549
	APP	471.2 ± 0.1581	287.66 ± 0.1581
	RP	296.1 ± 0.1581	360.08 ± 0.1549

APA, *Acyrthosiphon pisum*-alfalfa race; APP, *Acyrthosiphon pisum-*pea race; SA, *Sitobion avenae;* RP, *Rhopalosiphum padi* (In dark the natal host).

Bold numbers indicate time to first behaviour on natal host as baseline comparison.

* Parasitoids did not perform this behavior on the assayed host.

#### Sting behavior

**Figure 3:**
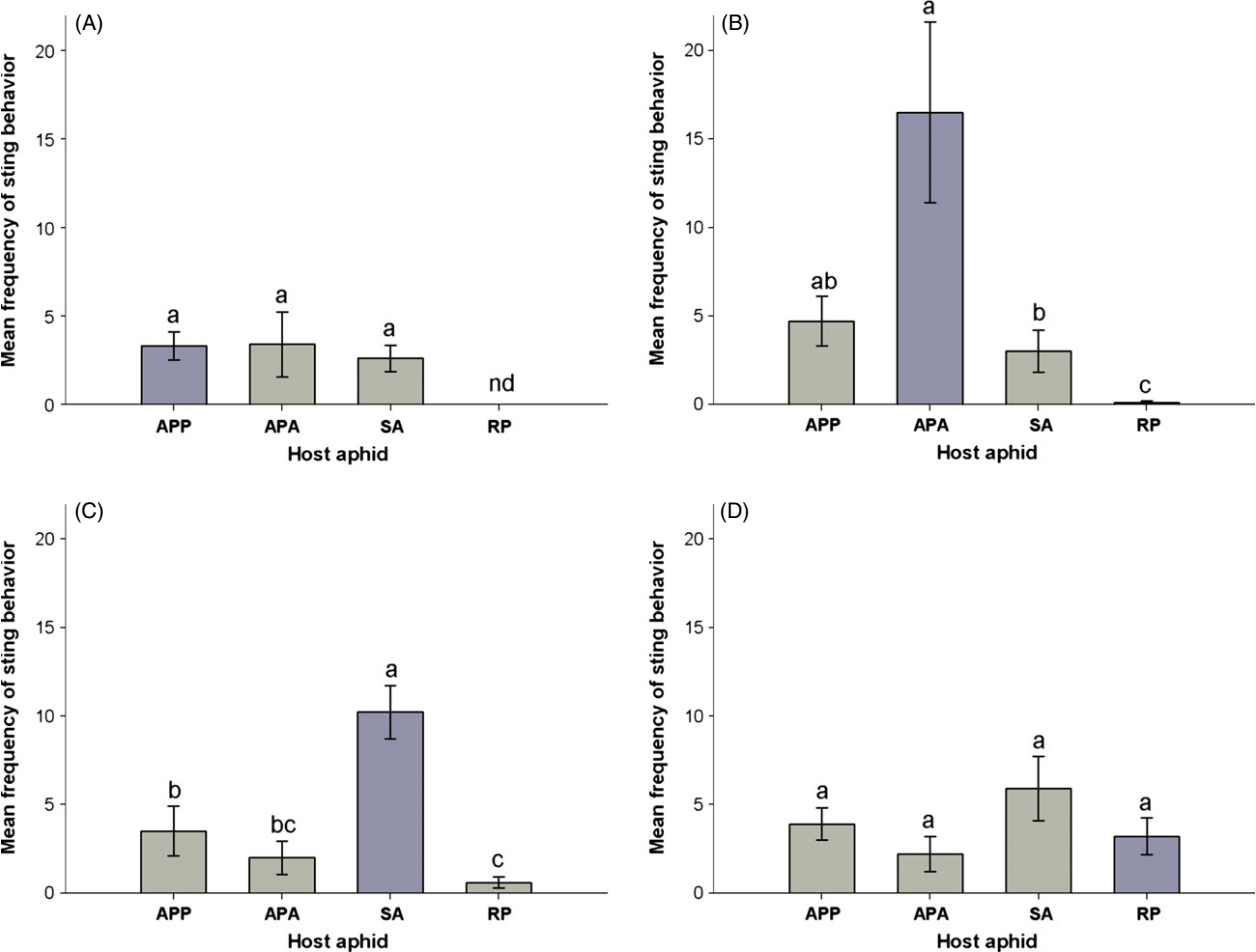
Mean frequency of *stings* of each parasitoid population on natal host (dark bars) and on non-natal hosts (light bars) (Mean ± SE). Different letters indicate significant differences expressed per parasitoid population assayed on the four hosts tested; *Acyrthosiphon pisum*-pea race (APP), *Acyrthosiphon pisum*-alfalfa race (APA), *Sitobion avenae* (SA), and *Rhopalosiphum padi* (RP). Figure: Parasitoid collected from (A) *A. pisum*-pea race; (B) *A. pisum*-alfalfa race; (C) *S. avenae* and (D) *R. padi*. nd; denotes no data.

**Figure 4:**
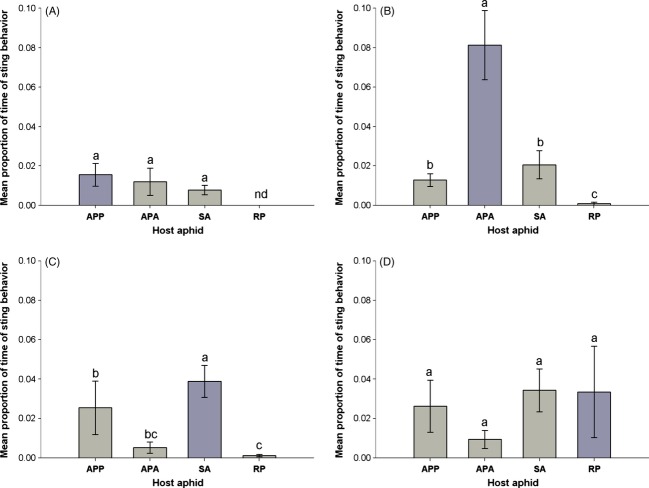
Mean proportion of time spent stinging natal host (dark bars) and non-natal hosts (light bars) of each parasitoid population (Mean ± SE). Different letters indicate significant differences expressed per parasitoid population assayed on the four hosts tested; *Acyrthosiphon pisum*-pea race (APP), *Acyrthosiphon pisum*-alfalfa race (APA), *Sitobion avenae* (SA), and *Rhopalosiphum padi* (RP). Figure: Parasitoid collected from (A) *A. pisum*-pea race; (B) *A. pisum*-alfalfa race; (C) *S. avenae* and (D) *R. padi*. nd: denotes no data.

A significant effect of the frequency and proportion of time spent stinging was observed for the *A. pisum*-alfalfa race and *S. avenae-*originated parasitoid populations tested on the assayed hosts (Table 1). Both populations showed a higher mean proportion of time of stinging on their natal host compared to all non-natal hosts. However, only parasitoids from *S. avenae* showed a high frequency of sting on their natal host compared with all non-natal hosts (Figs 3 and 4). Significant differences were also observed for the time to the first sting on the assayed hosts for three of the four parasitoid populations studied (APA, APP, and SA), being quicker on their natal hosts. No significant differences were observed for the time to first sting for the *R. padi-*originated parasitoid population between the assayed hosts (Table 2); (APA parasitoid population: Likelihood ratio test = 16.76, *P* = 0.0007; logrank test = 13.99, *P* = 0.002; df = 3); (APP parasitoid population: Likelihood ratio test = 20.07, *P* = 0.0001; logrank test = 16.43, *P* < 0.001; df = 3); (SA parasitoid population: Likelihood ratio test = 12.57, *P* < 0.01; logrank test = 15.77, *P <* 0.01; df = 3); (RP parasitoid population: Likelihood ratio test = 4.27, *P* = 0.234; logrank test = 4.15, *P =* 0.245; df = 3) (Table 2).

#### Walking behavior

**Figure 5:**
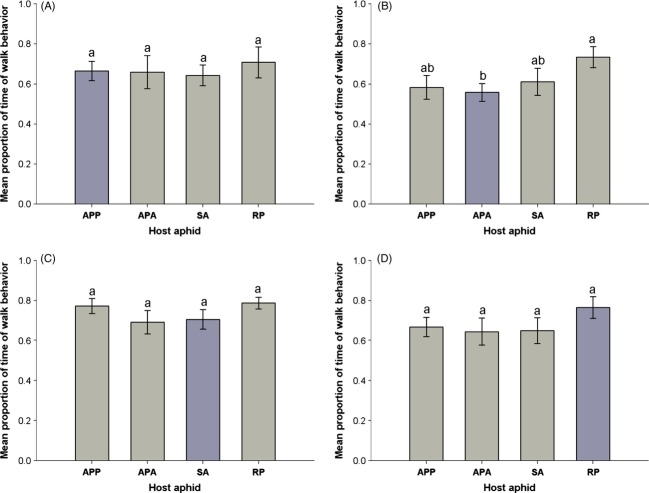
Mean proportion of time spent walking on natal host (dark bars) and non-natal hosts (light bars) of each parasitoid population (Mean ± SE). Different letters indicate significant differences expressed per parasitoid population assayed on the four hosts tested; *Acyrthosiphon pisum*-pea race (APP), *Acyrthosiphon pisum*-alfalfa race (APA), *Sitobion avenae* (SA), and *Rhopalosiphum padi* (RP). Figure: Parasitoid collected from (A) *A. pisum*-pea race; (B) *A. pisum*-alfalfa race; (C) *S. avenae* and (D) *R. padi*.

The proportion of time spent *walking* on hosts showed significant differences only for the *A. pisum*-alfalfa race-originated parasitoid population (Table 1), when *R. padi* was offered as a host they spent significantly more time walking around the experimental arena, compared to the natal host (Fig. 5). The remaining parasitoid populations studied here (APP, SA, and RP) did not show any significant difference among any of the tested hosts (Fig. 5).

### Virulence to natal and non-natal hosts

#### Parasitism rates

**Figure 6:**
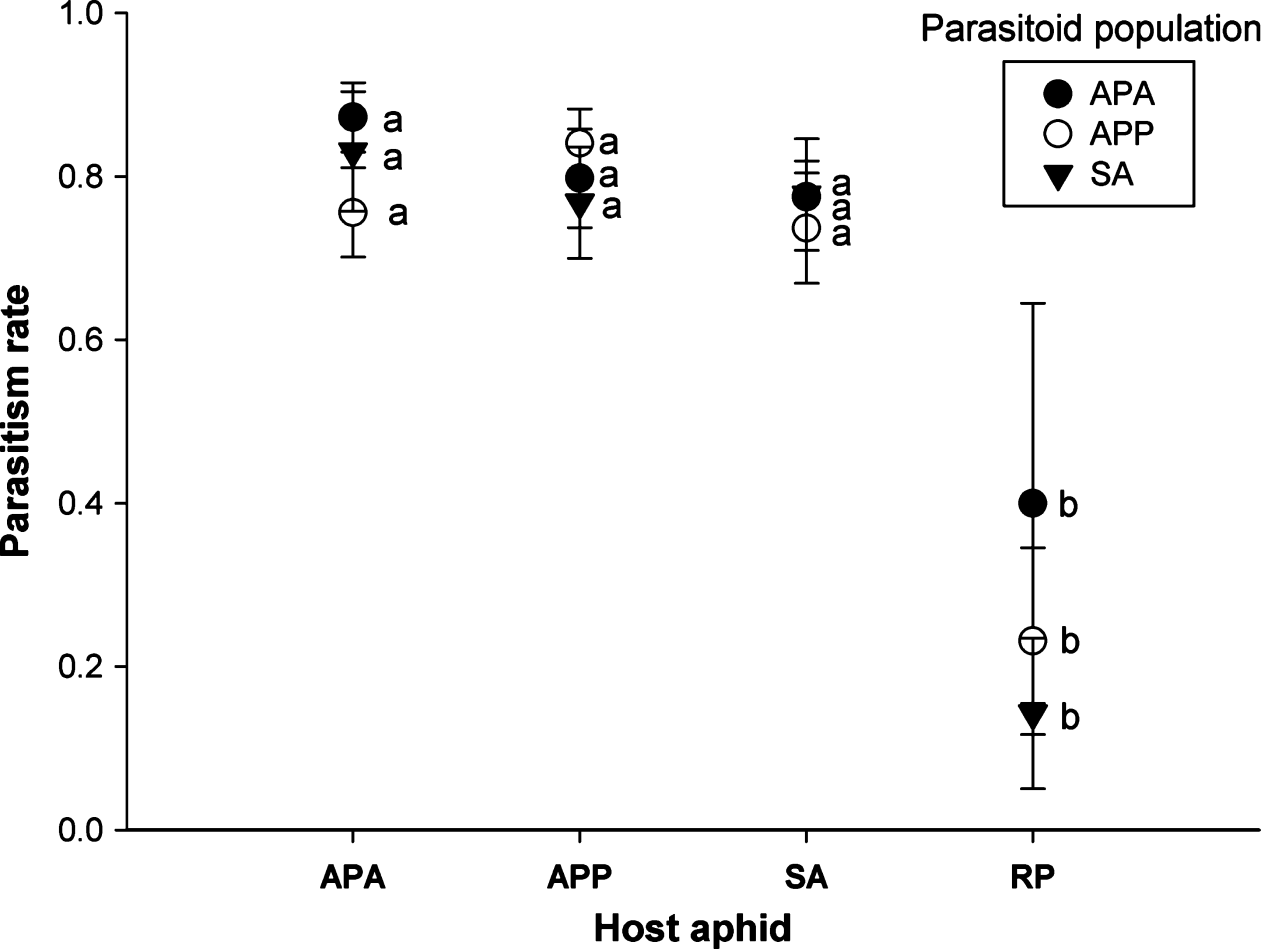
Parasitism rate; proportion (mean ± SE) of aphid mummies formed by parasitoid females with different host origins on their natal and non-natal hosts. Assayed hosts and parasitoids origins were: *Acyrthosiphon pisum*-alfalfa race (APA); *Acyrthosiphon pisum*-pea race (APP); *Sitobion avenae* (SA); and *Rhopalosiphum padi* (RP).

The parasitism rate was studied through quantifying the mean number of mummies formed from the stung nymphs (Fig. 6). A significant effect of the assayed host was observed (GLMM: assay hosts, χ^2^ = 46.29, df = 3, *P* < 0.001). This effect was caused by the very low parasitism rate observed on the *R. padi* host (0.24 ± 0.08: mean parasitism rate ± standard error), compared with a high parasitism rate observed on the *A. pisum*-alfalfa race (0.83 ± 0.03), the *A. pisum*-pea race (0.81 ± 0.03), and *S. avenae* (0.77 ± 0.03). Additionally, and due to these outcomes, it was not possible to analyze survival, productivity, sex ratio, or the developmental time of *R. padi* aphids as hosts. No significant effects in parasitism rates for the parasitoid population factor were found (GLMM: parasitoid population, χ^2^ = 2.04, df = 2, *P* = 0.3613). Nor were they found for the interaction between factors (GLMM: assay hosts x parasitoid population, χ^2^ = 9.81, df = 6, *P* = 0.1331).

#### Survival of the progeny

**Figure 7:**
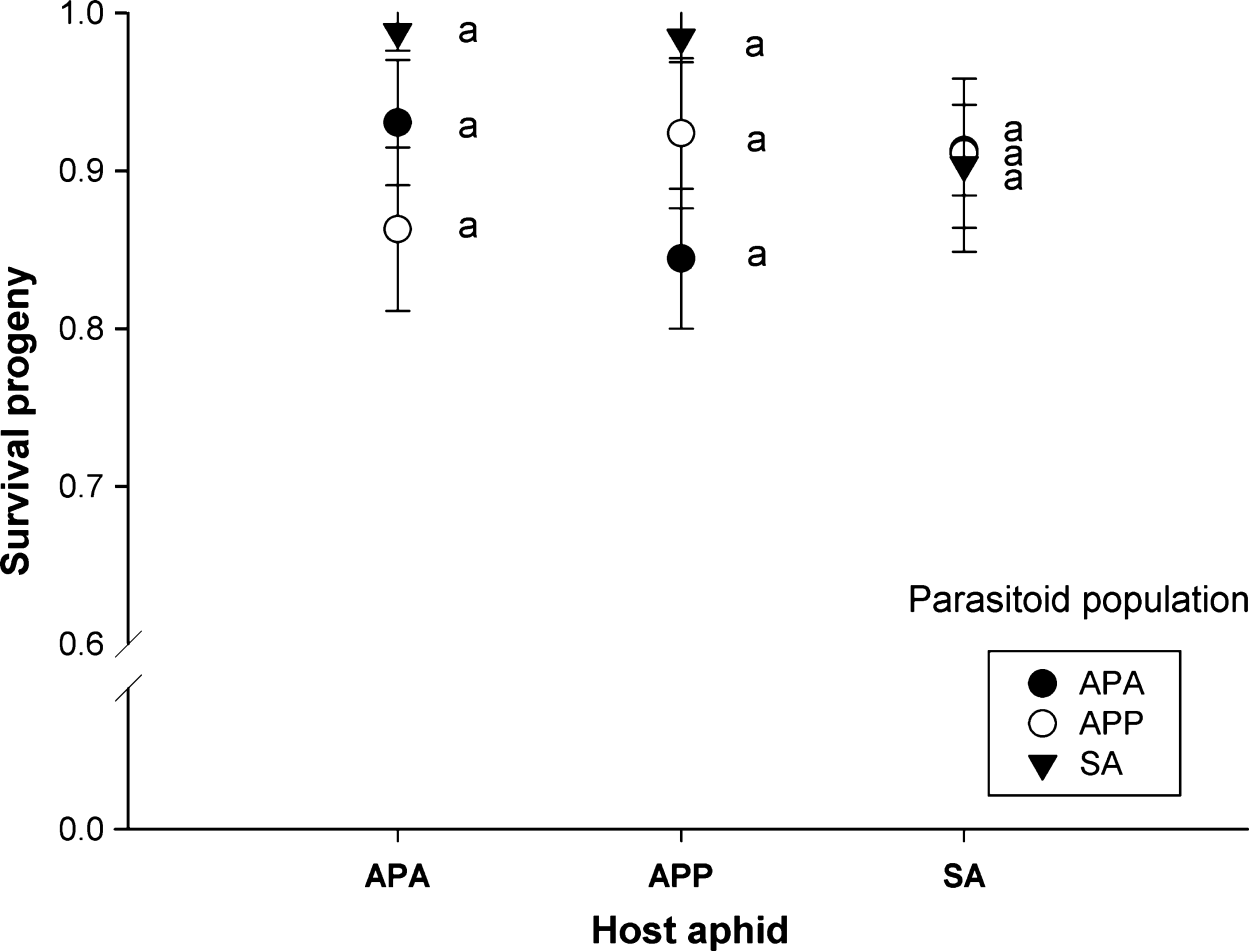
Survival; proportion (mean ± SE) of adult emergence of parasitoid progeny from assayed hosts. Assayed hosts and parasitoids origins were: *Acyrthosiphon pisum*-alfalfa race (APA); *Acyrthosiphon pisum*-pea race (APP); *Sitobion avenae* (SA); and *Rhopalosiphum padi* (RP).

The survival of the progeny was studied as the mean proportion of parasitoids emerged from the formed mummies. No significant effect was found on the mean survival of the parasitoid progeny for the parasitoid population (GLMM: parasitoid population, χ^2^ = 2.83, df= 2, *P* = 0.2427), the assayed host (GLMM: assay hosts, χ^2^ = 0.42, df = 2, *P* = 0.8121), or for the interaction between these two factors (GLMM: assay hosts x parasitoid population, χ^2^ = 8.23, df = 4, *P* = 0.0836). The mean proportion of the progeny survival from parasitoid females coming from different natal hosts was in general high on the different hosts assayed (more than 0.9 of mean survival) (Fig. 7).

#### Productivity

**Figure 8:**
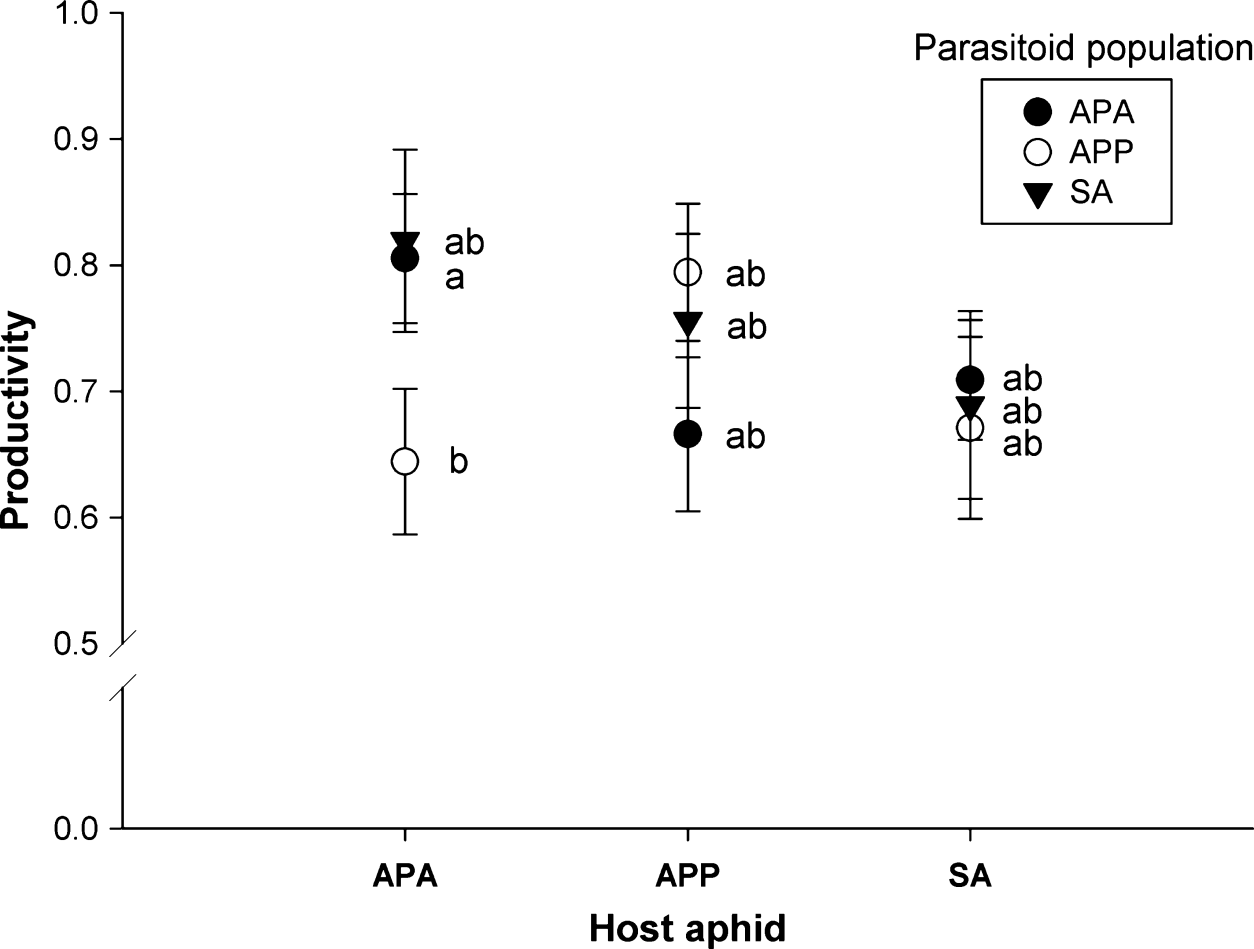
Productivity (mean ± SE) of the *A. ervi* populations with different host origins on the assayed hosts. Assayed hosts and parasitoids origins were: *Acyrthosiphon pisum*-alfalfa race (APA); *Acyrthosiphon pisum*-pea race (APP); *Sitobion avenae* (SA); and *Rhopalosiphum padi* (RP).

A significant effect of the interaction between factors was observed on productivity (GLMM: assay hosts x parasitoid population, χ^2^ = 14.81, df = 4, *P* < 0.01). Productivity was significantly different when comparing parasitoids from the *A. pisum*-alfalfa race on their natal host (APA: 0.81 ± 0.05) to the low productivity showed by parasitoids from the *A. pisum*-pea race (APA: 0.64 ± 0.06), when assayed on *A. pisum*-alfalfa race (Fig. 8). Moreover, the parasitoids from *S. avenae* showed no significant differences for productivity on their natal host (SA: 0.7 ± 0.07) compared to the non-natal hosts (APA: 0.82 ± 0.07 and APP: 0.76 ± 0.07) (Fig. 8). There were no significant effects for the assayed host (GLMM: assay hosts, χ^2^ = 1.86, df = 2, *P* = 0.3941) or for the parasitoid population (GLMM: parasitoid population, χ^2^ = 2.84, df = 2, *P* = 0.2419).

#### Sex ratio

The sex ratio of the progeny of parasitoid females developed on natal and non-natal hosts was studied. A significant effect was observed for the assayed host (GLMM: assay hosts, χ^2^ = 6.05, df = 2, *P* < 0.05), but not for the parasitoid population (GLMM: parasitoid population, χ^2^ = 0.06, df = 2, *P* = 0.9709). Significant differences in the sex ratio of the parasitoids were observed between the progeny originated from the *A. pisum*-alfalfa race and *S. avenae*. The progeny of the female parasitoids, irrespective of the source host (all different parasitoid populations) developed on *A. pisum*-alfalfa host race, showed a male-biased sex ratio (0.68 ± 0.05; mean male proportion in the total progeny ± standard error of the mean), which was significantly different from that observed on the *S. avenae* host, where the sex ratio was closer to equality (0.56 ± 0.05). Otherwise, no significant differences in sex ratios of the progeny originated from the *A. pisum*-alfalfa and the *A. pisum*-pea races (0.61 ± 0.05) were observed. The interaction between the factors was significant (GLMM: assay hosts x parasitoid population, χ^2^ = 10.17, df = 4, *P* < 0.05). Parasitoids from the *A. pisum*-alfalfa race showed a male-biased sex ratio on their natal host (APA: 0.75 ± 0.06), when compared to the non-natal host race, where a significant closer-to-equality sex ratio was observed (APP: 0.47 ± 0.08) (Data S1). In addition, there were no significant differences among sex ratios in parasitoids from *S. avenae* on the different hosts assayed, although it is worth noting that these exhibited a close to equality sex ratio on their natal host (SA: 0.48 ± 0.08), showing marginally significant differences when comparing the sex ratio expressed on non-natal hosts (APA: 0.67 ± 0.1 and APP: 0.65 ± 0.09).

#### Developmental time

Parasitoids from different hosts showed no significant differences in their mean developmental time on the different assayed hosts, ranging from 15.0 ± 0.19 to 17.2 ± 0.8 days (mean number of development days ± standard error). No significant effects were found for the analyzed factors or for the interaction between these factors (GLMM: assay hosts, χ^2^ = 4.28, df = 2, *P* = 0.1177; parasitoid population, χ^2^ = 0.56, df = 2, *P* = 0.7541; assay hosts × parasitoid population, χ^2^ = 0.39, df = 4, *P* = 0.9825) (data not shown).

### Prevalence of *Aphidius ervi* on different aphid hosts in the field

Field sampling revealed that *A. ervi* is the most common parasitoid species parasitizing the *A. pisum* complex. It represents more than 94% of all parasitoid individuals emerging from this host in the two different regions sampled (in total 877 individuals) (Data S2). Additionally, the diversity of parasitoid species associated with *A. pisum* was low, including parasitoids from the genus *Praon* (*P. volucre* and *P. gallicum*) and *Aphidius* (*A. matriariacae* and *A. colemani*). All these species have been described as parasitoids prone to be habitat generalists (associated with different plant families) and host aphid generalists (Starý et al. 1994). On cereal aphids, a larger parasitoid diversity was observed in comparison with that observed on the *A. pisum* complex. A total of 596 parasitoid individuals were sampled from the aphid *S. avenae,* identifying up to nine parasitoid species, the most represented being the genus *Aphidius*. These included *A. ervi* (38%)*, A. uzbekistanicus* (28%), *A. rhopalosiphi* (12%), *A. picipes* (3%), *A. colemani* (3%), and *A. matricariae* (0.4%), although *P. volucre* (9%)*, P. gallicum* (6%), and *Lysiphlebus testaceipes* (0.5%) were also detected. Furthermore, *S. avenae* and *R. padi* appeared as the common resource for the same parasitoid assemblage. From the aphid *R. padi,* a total of 250 parasitoid individuals were obtained. *R. padi* was mostly parasitized by *A. colemani* (34%), *A. uzbekistanicus* (22%), *A. rhopalosiphi* (20%) and by *A. ervi* to a lesser extent (11%), but also by *L. testaceipes* (10%), *A. picipes* (2.4%), *P. volucre* (0.4%), *P. gallicum* (0.4%), and *A. matricariae* (0.4%) (Data S2). These observations are in agreement with what has been previously reported by Starý et al. (1994), Gerding et al. (1989) and Gerding and Figueroa (1989), who described *A. ervi* as the most predominant parasitoid species controlling *S. avenae* aphids in Chile.

## Discussion

Parasitoid insects represent one of the most used natural enemies for biological pest control as they are commonly considered to be host-specific (Godfray 1994). Not only does this mean that the released parasitoids will be most efficient at attacking the target pest species, but also it reduces the possibility of environmental harm through spillover of rapidly growing populations from crops into adjacent natural habitats (Rand et al. 2006), as has been observed for generalist predators (Duelli et al. 1990). However, parasitoids are seldom specific to the point of attacking only one host species, and there are many parasitoid species that have a wide host range (Mackauer and Starý 1967). Also, the parasitoid host range may not be consistent across the distribution of an entire species, and different studies have reported the possibility of host adaptation occurring in parasitoid populations, thus restricting the potential host range (Antolin et al. 2006; Stireman et al. 2006; Abrahamson and Blair 2008; Henry et al. 2008). Indeed, within the range of all potential hosts, not all of them are equally preferred and/or become susceptible to the development of parasitoids (Desneux et al. 2009).

### Host range use by *Aphidius ervi*

The parasitoid *A. ervi* has been described not only as a habitat generalist, but also as a moderate host specialist, being found associated with different crops (e.g., poaceas, fabaceas, and solanaceous). However, it is not necessarily parasitic to all available host species associated with those crops (Stilmant et al. 2008). For instance, the aphid *R. padi* has been reported within the range of potential hosts for *A. ervi* (Kavallieratos et al. 2004). In this context, the reciprocal transplant experiments conducted in this study showed that *A. ervi* females are able to discriminate and choose between host aphid species that are experimentally offered for oviposition. In fact, the results indicate that parasitoids collected from *A. pisum*-alfalfa race and *S. avenae* showed the lowest infectivity on *R. padi* (Figs 3 and 4). Moreover, *A. pisum* (both host races), *S. avenae*, and even *R. padi-*originated parasitoids were characterized by taking more time to achieve a first sting when *R. padi* was offered as a host, which could be interpreted as a nonpreference (Table 2). Furthermore, *A. pisum-*pea race-originated parasitoids did not perform any stinging behavior on *R. padi*. At the same time, these behaviors were accompanied by a reduced parasitism rate on *R. padi* aphids compared with the other assayed hosts *(A. pisum* complex and *S. avenae*), thereby suggesting that *R. padi* represents a low-quality host for *A. ervi*. This was corroborated by the lower prevalence of *A. ervi* on *R. padi* observed in the field, in comparison with the higher prevalence on *A. pisum* and *S. avenae*. Recently, Desneux et al. (2012) suggested that host range constraints among parasitoids could be strongly influenced by host phylogeny (phylogeny specialization). Therefore, closely related host aphid species are more likely to share traits related to susceptibility to a certain parasite or parasitoid due to a common evolutionary history (Desneux et al. 2012). This hypothesis has been proposed for the aphid-parasitoid *Binodoxys communis*, for which a strong effect of host phylogeny on host acceptance (preference) and parasitoid survival (performance) was described, showing that both traits showed a phylogenetic conservatism with respect to the host species (Desneux et al. 2009). *Binodoxys communis* successfully parasitizes hosts with a closer phylogenetic proximity to its main host, the soybean aphid *Aphis glycines,* rather than other phylogenetically more distant hosts (Desneux et al. 2012). In this sense, the results reported here show that *A. ervi* maintains a higher infectivity and virulence on the *A. pisum* and *S. avenae* hosts, which indeed have a closer phylogenetic proximity (both belonging to the tribe Macrosiphini). Consequently, the lower infectivity and virulence (parasitism rate) observed in the phylogenetically more distant *R. padi* (belonging to the tribe Aphidini) supports the Desneux et al. (2012) hypothesis as both tribes diverged over 50–70 million of years ago (von Dohlen et al. 2006). Nevertheless, further studies are necessary to determine whether this pattern of host use observed for *A. ervi* is influenced by host phylogeny, including several populations and throughout different geographical ranges.

### Pattern of local adaptation in *Aphidius ervi* parasitoids: infectivity and virulence on natal and non-natal hosts

The use of different hosts imposes divergent selection pressures on parasitoid populations through fitness trade-offs related to the use of different hosts, thus driving local adaptation of parasitoid populations to their natal host (Kawecki and Ebert 2004). Several interacting factors may counteract the adaptive process, including gene flow, environmental variability, phenotypic plasticity, and the lack of genetic diversity (Kawecki and Ebert 2004; Crispo 2008). The results obtained in this study indicate that natural populations of *A. ervi* coming from different hosts in the field exhibit important differences in infectivity on their natal host in comparison with non-natal hosts, although this pattern was not observed for all the parasitoid populations nor in all behavioral variables studied. Despite the above, the virulence (a proxy for fitness in parasitoids) expressed by these populations on the tested aphid hosts shows a lack of local host adaptation. Parasitoids obtained from the aphid hosts *S. avenae*, *A. pisum*-alfalfa race, and *A. pisum*-pea race showed a greater infectivity on their natal host in comparison with that shown on non-natal hosts, but only for some of the behavioral variables studied (e.g., time to first sting). For parasitoid populations from *S. avenae* and *A. pisum*-alfalfa race, the proportion of time spent stinging was significantly greater when the natal host was offered compared to non-natal hosts, and for the *S. avenae*-originated parasitoids, the frequency of stinging was significantly greater when the natal host was offered compared to non-natal hosts. Otherwise, significant differences in ‘the time to the first attack’ on hosts were observed for parasitoid populations from *S. avenae* and *A. pisum*-pea race, while significant differences for ‘the time to the first sting’ on hosts were observed for three of the four parasitoid populations studied (from both *A. pisum* races and *S. avenae*), taking less time to attack and sting when the natal host was offered in comparison with the non-natal hosts. Contrastingly, the virulence assay showed a high plasticity for traits related to fitness. The three different parasitoid populations studied (from both *A. pisum* races and *S. avenae*) showed a similar high virulence (parasitism rate, survival, and productivity) on natal and non-natal hosts (APA, APP, and SA), thus providing evidence for the absence of local host adaptation. Additionally, an unusual male-biased sex ratio in the offspring of parasitoid females on both natal and non-natal hosts was observed, as well as an unexpected male-biased sex ratio of parasitoids from the *A. pisum*-alfalfa race on their natal host when compared to the non-natal host race. A possible explanation for these finding is that aphids from early stages were used for these assays, and inadvertently, some variation in size of the aphids used could have affected, to some degree, the female sex allocation. The theory of sex allocation predicts that parasitoids should lay male eggs in small hosts and female eggs in large hosts (Charnov et al. 1981; Godfray 1994).

The results presented here do not support the ‘sequential radiation’ hypothesis for these introduced populations (Abrahamson and Blair 2008), as parasitoids collected from both host races of *A. pisum* did not show fitness compromises in the reciprocal transplant experiments (i.e., a higher fitness on their natal host race than on non-natal host races). Consequently, Bilodeau et al. (2013) rejected the sequential radiation hypothesis for introduced populations of *A. ervi* in North America, based on population genetic and experimental data. The inconsistency between the lack of local host adaptation and the differences in parasitoid infectivity observed is not surprising. A joint evolution of preference and performance is not always observed, due to the need for coupling based on pleiotropy or by linkage disequilibrium between both traits (Futuyma and Moreno 1988; Forister et al. 2007). In addition, unlike the traits related to the parasitoid virulence, behavioral traits related to parasitoids infectivity can be strongly influenced by imprinting effects (i.e., host fidelity due to the preadult-host experience), thus explaining the greater preference to the host from which they emerged. Different studies have demonstrated that host fidelity in *A. ervi* can be induced even after a single generation, indicating that this mechanism is plastically induced and not under direct selection (Daza-Bustamante et al. 2002; Henry et al. 2008). Although host fidelity has proved to encourage the host adaptation through continual use of the same host species (Forbes et al. 2009), by itself it is not sufficient for the formation and maintenance of host adaptations in parasitoid populations, even under reduced gene flow between populations. Fitness trade-offs on the alternate hosts in these populations is a critical prerequisite for local adaptation (Kawecki and Ebert 2004; Abrahamson and Blair 2008). In this regard, the lack of local host adaptation in our results could be explained by the selective environments where *A. ervi* is found, where, in rapidly changing environments, generalist plastic phenotypes would be favored. In fact, the evolution of adaptive phenotypic plasticity (i.e., plasticity that increases mean fitness across environments) could be favored over local adaptation in the presence of environmental heterogeneity (Kawecki and Ebert 2004; Crispo 2008; Svanbäck et al. 2009). In this respect, the aphid-parasitoid population dynamics in agroecosystems has been classically described as a metapopulation, characterized by frequent local extinctions and recolonizations (Weisser 2000; Rauch and Weisser 2007). In doing so, frequent extinctions obliterate locally adapted gene pools through increased dispersal, so extinction-colonization dynamics (i.e., metapopulation dynamics) are unfavorable to local adaptation (Kawecki and Ebert 2004). Thus, fluctuations in host abundance (in time and/or space) could favor the maintenance of generalist parasitoid populations with the ability to simultaneously maximize fitness on more than one host (Hufbauer and Roderick 2005). However, other nonmutually exclusive explanations must not be ruled out. For instance, a short time has elapsed since the introduction of *A. ervi* in Chile, so the potential loss of genetic variation during the introduction process could have delayed the adaptive process. Furthermore, a high gene flow of *A. ervi* among other aphid hosts could be detrimental to local host adaptation, due to its homogenizing effect of the genetic variation between parasitoid populations through a continuous gene introgression within locally adapted demes (Rasanen and Hendry 2008). In the same sense, a high gene flow could also result during the selection for increased plasticity, or alternatively, plasticity may promote gene flow between different selective regimens (Crispo 2008) by allowing migrants to maintain high fitness (virulence) across alternative hosts and thus facilitate colonization, population growth, and population persistence (Thibert-Plante and Hendry 2010; Chevin and Lande 2011), which would translate into a direct benefit for the control of a target pest.

### Implications for biological control

Regarding the use of alternative hosts, different evolutionary trajectories could be followed by biological control agents after their introduction to new geographic areas. In this way, natural, locally adapted enemies should have higher rates of population increase and/or be more efficient at controlling a certain aphid host (Hufbauer and Roderick 2005). However, high gene flow rates between host-associated populations could prevent local adaptation, while adaptive phenotypic plasticity may be favored over adaptive differentiation in organisms living in fluctuating environments (Thibert-Plante and Hendry 2010) such as most agricultural environments. Our results have shown the lack of local host adaptation and highlight the role of phenotypic plasticity allowing parasitoids to maximize fitness on more than one host, thus enabling them to potentially use different aphid species. This observation is accompanied by evidence of a high gene flow among different host-associated parasitoid populations in the field in Chile (F. A. Zepeda-Paulo, unpublished). However, the parasitoid *A. ervi* does not equally use its potential host range, showing a low preference and virulence to *R. padi*, which suggests an effect of host phylogeny on the traits studied. In addition, the infectivity of *A. ervi* shows host preferences mediated through host fidelity of some populations, demonstrated by significant differences in infectivity across the different hosts studied. In this respect, host fidelity has been observed to have an effect on parasitism rates in mass-reared *A. ervi* on a novel host; however, these preferences could be switched back to the original host in a single generation (Henry et al. 2008). In terms of pest suppressiveness, a highly plastic control agent such as *A. ervi* should be more suppressive in field crops than other more rigid and specialized parasitoids because they would be more resilient to environmental changes, thus increasing any insurance effects. Therefore, changing agricultural settlings, characterized by frequent crop rotations between seasons that include different plant families and short crop cycles, will result in variations in aphid biodiversity and abundance on alfalfa and gramineous crops (or after pesticide use and tillage). The latter could particularly affect locally adapted parasitoid populations by reducing the resilience of such landscapes. Hence, introduced biocontrol agents with the capacity to use and maintain a high performance on alternative hosts could allow a more efficient biological control through the continuous movement of parasitoids from alternative hosts acting as reservoirs to the target pest populations (Starý 1993; Landis et al. 2000). In fact, the simultaneous control of a target and nontarget pest by the same parasitoid biocontrol agent has been proposed as the key factor for the successful biocontrol of cereal aphids in Chile (Starý et al. 1993). On the other hand, high parasitoid plasticity in the use of distinct hosts could be favored (or adaptive) under the current climate-change scenario, which predicts a greater frequency and intensity of pest outbreaks due to the disruption of parasitoid–herbivore dynamics (Stireman et al. 2005; Tylianakis et al. 2008). Indeed, a genotype–genotype association has been shown to be altered by the recent increase in local average temperatures in an aphid-parasitoid association of a cultivated crop (Lavandero and Tylianakis 2013). All this poses the need to incorporate new eco-evolutionary approaches in the selection process of biocontrol agents. In addition, our present study reaffirms the usefulness of this experimental approach to study patterns of adaptation in biocontrol agents to certain target hosts, thus making a clear distinction between infectivity (preference) and virulence (proxy of fitness) of parasitoids, because often both are camouflaged in the measures of adaptation or explicitly focused on infectivity as the main measure of host adaptation (Hufbauer and Roderick 2005). Future research should be focused on the potential of phenotypic plasticity as an adaptive mechanism in generalist parasitoids living in changing environments, determining the effect of high plastic parasitoids on the efficiency of pest control, and quantifying the relative frequency and dynamics of these *A. ervi* and aphid-host populations in the field. This will become especially relevant, as practices of biological control will need to adopt new strategies for choosing agents and their release under the present climate change (Roderick et al. 2012). The role of endosymbionts should not be neglected either as, most likely, they have an important role to play in the abundance and efficacy of biological control agents in the field and their prevalence.

## Data archiving statement

Data deposited in the Dryad repository: doi:10.5061/dryad.5735t.

## Supporting Information

**Data S1.** Sex ratio (mean ± SE) of the emerging progeny (proportion of males) of parasitoid females with different host origins on assayed hosts. Horizontal lines represent a ratio of gender equality (0.5). The assayed host and parasitoids origin were: *A. pisum*-alfalfa race (APA); A. *pisum*-pea race (APP); *S. avenae* (SA) and *R. padi* (RP).

**Data S2.** Prevalence (% of parasitoid individuals) of different parasitoid species collected in Chile on *Acyrthosiphon pisum, Sitobion avenae* and *Rhopalosiphum padi* in A) Region del Maule and B) Region de Los Rios.
