# Supplementary figures and images for: Adaptive evolution of a generalist parasitoid: implications for the effectiveness of biological control agents
Source: Evol Appl. 2013 Aug 5;6(6):983–99. doi: 10.1111/eva.12081 (PMC3779098; doi:10.1111/eva.12081)

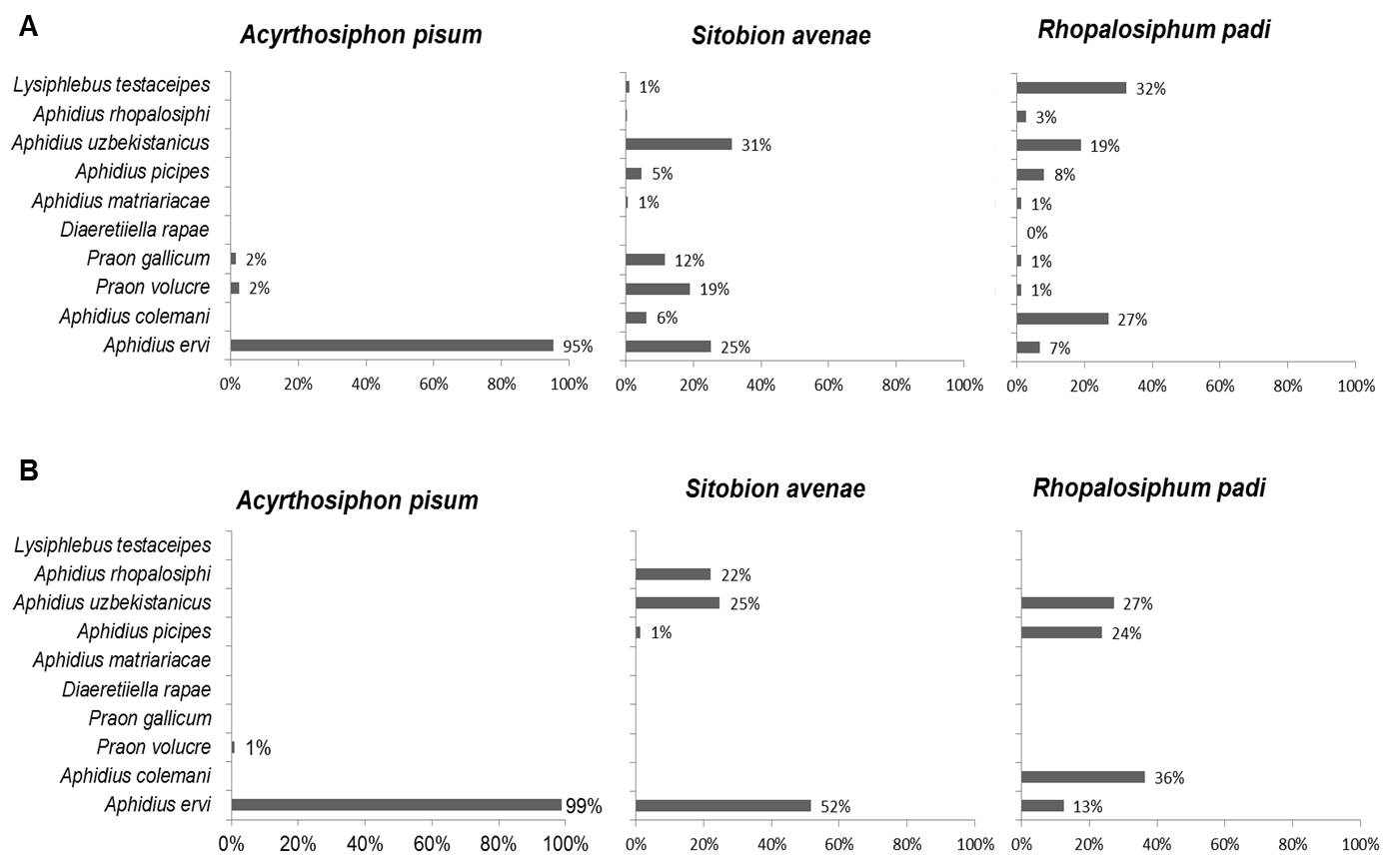

Supplement: Supplementary file 2 [file eva0006-0983-SD2.tif]
