# Supplementary material for: Adaptive evolution of a generalist parasitoid: implications for the effectiveness of biological control agents
Source: Evol Appl. 2013 Aug 5;6(6):983–99. doi: 10.1111/eva.12081 (PMC3779098; doi:10.1111/eva.12081)
Supplement: Supplementary file 3 [file eva0006-0983-SD3.doc]

**Supplementary material legends**

**Data S1** Sex ratio (mean ± SE) of the emerging progeny (proportion of males) of parasitoid females with different host origins on assayed hosts. Horizontal lines represent a ratio of gender equality (0.5). The assayed host and parasitoids origin were: *A. pisum*-alfalfa race (APA); *A. pisum*-pea race (APP); *S. avenae* (SA) and *R. padi* (RP).

**Data S2** Prevalence (% of parasitoid individuals) of different parasitoid species collected in Chile on *Acyrthosiphon pisum, Sitobion avenae* and *Rhopalosiphum padi* in A) Region del Maule and B) Region de Los Rios.
